# Detection of (pre)cancerous colorectal lesions in Lynch syndrome patients by microsatellite instability liquid biopsy

**DOI:** 10.1038/s41417-023-00721-z

**Published:** 2024-02-09

**Authors:** Mattia Boeri, Stefano Signoroni, Chiara Maura Ciniselli, Manuela Gariboldi, Susanna Zanutto, Emanuele Rausa, Miriam Segale, Anna Zanghì, Maria Teresa Ricci, Paolo Verderio, Gabriella Sozzi, Marco Vitellaro

**Affiliations:** 1 Epigenomics and Biomarkers of Solid Tumors Unit, Experimental Oncology Department, Fondazione IRCCS Istituto Nazionale dei Tumori, Milan, Italy; 2 Unit of Hereditary Digestive Tract Tumors, Department of Surgery, Fondazione IRCCS Istituto Nazionale dei Tumori, Milan, Italy; 3 Bioinformatics and Biostatistics Unit, Fondazione IRCCS Istituto Nazionale dei Tumori, Milan, Italy; 4 Molecular Epigenomics Unit, Fondazione IRCCS Istituto Nazionale dei Tumori, Milan, Italy; 5 Colorectal Surgery Division, Department of Surgery, Fondazione IRCCS Istituto Nazionale dei Tumori, Milan, Italy

**Keywords:** Biomarkers, Cancer prevention

## Abstract

Lynch syndrome (LS) is an inherited condition characterized by an increased risk of developing cancer, in particular colorectal cancer (CRC). Microsatellite instability (MSI) is the main feature of (pre)cancerous lesions occurring in LS patients. Close endoscopic surveillance is the only option available to reduce CRC morbidity and mortality. However, it may fail to intercept interval cancers and patients’ compliance to such an invasive procedure may decrease over the years. The development of a minimally invasive test able to detect (pre)cancerous colorectal lesions, could thus help tailor surveillance programs in LS patients. Taking advantage of an endoscopic surveillance program, we retrospectively assessed the instability of five microsatellites (BAT26, BAT25, NR24, NR21, and Mono27) in liquid biopsies collected at baseline and possibly at two further endoscopic rounds. For this purpose, we tested a new multiplex drop-off digital polymerase chain reaction (dPCR) assay, reaching mutant allele frequencies (MAFs) as low as 0.01%. Overall, 78 plasma samples at the three time-points from 18 patients with baseline (pre)cancerous lesions and 18 controls were available for molecular analysis. At baseline, the MAFs of BAT26, BAT25 and NR24 were significantly higher in samples of patients with lesions but did not differ with respect to the grade of dysplasia or any other clinico-pathological characteristics. When all markers were combined to determine MSI in blood, this test was able to discriminate lesion-bearing patients with an AUC of 0.80 (95%CI: 0.66; 0.94).

## Introduction

Lynch syndrome (LS) is an autosomal dominant hereditary condition characterized by an increased risk of developing several types of cancer, in particular colorectal cancer (CRC) [1]. Recent estimates report a prevalence of LS that can vary between 1:279 and 1:2000 [1–3].

Germline mutations or epimutations in DNA mismatch repair (MMR) genes such as *MLH1*, *MSH2*, *MSH6,* and *PMS2* have been identified as being responsible for this condition [4]. Once the remaining functional MMR allele is somatically inactivated, the acquired MMR deficiency enhances the progression of adenomas to more invasive tumors [5]. MMR deficiency is thus considered an early initiating event, which can in turn lead to the accumulation of numerous somatic mutations throughout the genome, including microsatellite instability (MSI), which is a peculiarity of precancerous and cancerous lesions in LS patients [5–7]. Many diagnostic tools based on polymerase chain reaction (PCR) and next-generation sequencing (NGS) technologies have been developed to evaluate MSI status in tumor tissue samples [8–11]. One of the most widely used methods interrogates five microsatellite markers (BAT25, BAT26, NR21, NR24, and Mono27): tumors displaying instability in two or more markers are classified as high-MSI [12, 13].

The risk of cancer in LS individuals, and subsequent surveillance recommendations, vary according to the altered MMR gene [7]. The overall estimated average risk of CRC in LS individuals is up to 14 times higher than in the general population and frequently manifests at younger ages, typically before age 50, with metachronous malignancies [14, 15]. Guidelines indicate that close endoscopic surveillance by colonoscopy every one to three years from a young age (20-30 years) on the basis of MMR gene mutational status and family history [7] is the only available option to reduce morbidity and mortality for LS patients [16, 17]. An important issue is that the compliance of patients to an invasive procedure like colonoscopy may decrease over the years, while the risk of developing cancer increases [5]. Other issues are overdiagnosis or, conversely, the failure of colonoscopy to intercept interval cancers occurring in between surveillance procedures [18].

In this complex scenario, the development of a minimally invasive test able to detect the presence of precancerous and early cancerous lesions would be crucial to stratify risks, tailor the surveillance program and increase patient acceptance. Liquid biopsy for the analysis of circulating tumor DNA (ctDNA) has emerged as a promising tool for diagnosis and monitoring treatment response in several cancer types including CRC [19–22]. Recently, Silveira et al. reported on the detection by digital PCR (dPCR) of MSI in liquid biopsies from patients diagnosed with CRC [23]. However, the efficacy of MSI detection in liquid biopsy for CRC surveillance in LS individuals has not been exploited to date.

Here we report the results of a retrospective study aimed at evaluating the utility of blood MSI (bMSI) to discriminate LS patients with and without precancerous or cancerous lesions enrolled in a CRC surveillance program.

## Methods

### Study design

Starting in 2013, LS patients with identified pathogenic germline mutations in MMR genes were enrolled in the INT03/13 observational study after giving their informed consent. The diagnosis of LS was carried out following genetic counseling, which considered aspects such as family and personal clinical history. Eligible patients were invited to undergo a genetic test to detect mutations and/or deletions in MMR genes on DNA extracted from peripheral blood. Only patients who carried a pathogenic (or likely pathogenic) germline variant were then diagnosed with LS. Enrolled patients underwent colonoscopy at baseline (T0) and were invited to return for a second (T1: 12-24 months after T0) and third (T2: 12–24 months after T1) screening round according to the standard surveillance guidelines. Plasma samples were collected from all patients at T0 and possibly at further time points. During each endoscopic examination, precancerous and cancerous lesions were identified and annotated in a dedicated database. Lesions were classified as low grade dysplasia adenoma (LgD), high-grade dysplasia adenoma (HgD), and three adenocarcinoma (ADK). For each patient, genetic, molecular, and clinical data as well as family history of cancer were also collected.

**Fig. 1:**
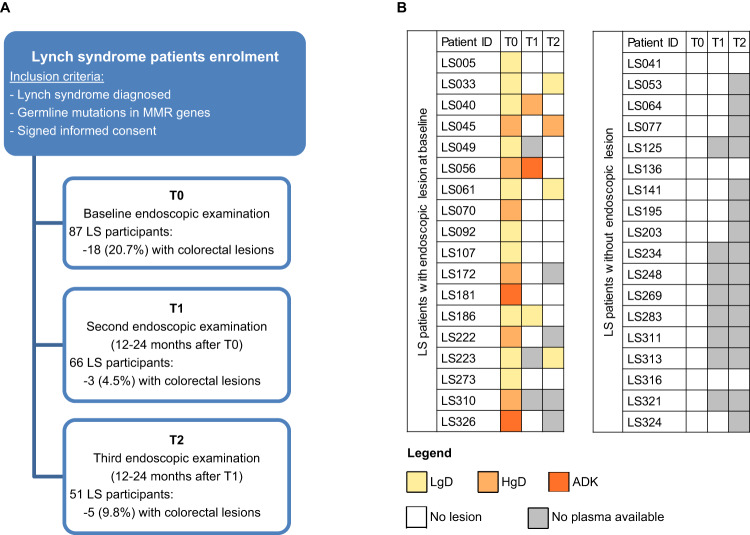
Characteristics of Lynch syndrome patients and selected plasma samples. **A** The flow chart of the colorectal cancer surveillance study enrolling Lynch syndrome patients and (**B**) the heat map of selected plasma samples used for dPCR analysis. The colors of the heat map reflect the presence and type of endoscopic lesions classified as low-grade dysplasia (LgD), high-grade dysplasia (HgD), or adenocarcinoma (ADK).

To evaluate the feasibility of MSI analysis in liquid biopsy in this exploratory study, we selected the 18 LS patients with lesions at T0 and an equal number of LS patients without lesions at baseline and at each subsequent surveillance round (Fig. 1). Patients were selected for the present study according to plasma availability at both T0 and subsequent time points. This study complied with the Declaration of Helsinki and was approved by the Ethics Committee of the Fondazione IRCCS Istituto Nazionale dei Tumori of Milan, Italy.

### Plasma sample collection and DNA extraction

Blood samples were collected in 10-mL Vacutainer tubes with spray-coated K_2_EDTA and the plasma was separated by double centrifugation at 1,258xg and 4°C for 10 minutes. The plasma samples were stored at -80°C in 1.5-mL cryovials until their use for this study. Circulating free DNA (cfDNA) was extracted from up to 2 mL of plasma using the Maxwell RSC ccfDNA plasma kit (Promega, Madison, WI, USA) according to the standard protocol and eluted in 50 μL of elution buffer. The cfDNA fraction was quantified by electrophoresis using the cfDNA ScreenTape Assay for TapeStation Systems (Agilent, Santa Clara, CA, USA) and data were analyzed using the TapeStation Analysis Software 4.1.1 considering 50-700 bp DNA fragments.

### DNA extraction and MSI status assessment in tumor tissue samples

Formalin-fixed paraffin-embedded (FFPE) 5-µm cut sections were manually microdissected to isolate the highest possible percentage of neoplastic cells. Tumor DNA was extracted using the Maxwell RSC DNA FFPE Kit. The extracted DNA was quantified using a NanoDrop 2000 (ThermoFisher, Waltham, MA, USA) spectrophotometer and related software. The MSI status of tumors was assessed by fluorescent pentaplex PCR of 5 microsatellites (Promega, Madison, WI, USA), as previously described [24]. To perform dPCR, the extracted tissue DNA was appropriately diluted to obtain a final concentration of 1–5 ng/µL.

### Molecular analysis by dPCR

The stability of the five microsatellite markers in plasma was analyzed using the multiplex drop-off MSI ddPCR Expert Design Assay (Bio-Rad, Hercules, CA, USA), the ProFlex 2× Flat PCR System (ThermoFisher, Waltham, MA, USA) and the QuantStudio 3D Digital PCR Instrument (ThermoFisher, Waltham, MA, USA). The Bio-Rad MSI probes are labeled with FAM or HEX fluorophores and compete to detect BAT25 and BAT26 (Assay 1 ID: dHsaEXD63918215), NR21 and NR24 (Assay 2 ID: dHsaEXD87594642) or Mono27 (Assay 3 ID: dHsaEXD12457761), batch number: 65800361. In the presence of the wild-type (WT) sequence, both probes bind the microsatellite marker, resulting in a double positive signal (FAM+/HEX+). Conversely, in the presence of the mutant sequence one of the probes drops off, resulting in a single positive signal (FAM-/HEX+ or FAM+/HEX−). Briefly, 15.8 µL of reaction mix containing 0.8 µL of 20X Bio-Rad MSI ddPCR assay, 8.4 µL of 2X QuantStudio 3D Digital PCR Master Mix and 6.6 µL of eluted cfDNA was prepared. All samples were loaded onto a 20,000-well chip using the self-moving chip loader according to the manufacturer’s instructions. Thermo-cycling was run in predefined amplification conditions: 96 °C for 10 min, 40 cycles at 55 °C for 1 min and 98 °C for 30 s, followed by an extension step at 60 °C for 2 min. Data were analyzed by two expert researchers using the QuantStudio 3D AnalysisSuite online tool (ThermoFisher, Waltham, MA, USA).

DNA from FFPE tumor tissue samples of two CRC patients with MSI was used as a positive control and elution buffer alone as a negative control. The controls served as a guide to manually assign scatter plot dots to the empty (FAM-/HEX-), WT (FAM+/HEX+), and mutant (FAM-/HEX+ or FAM+/HEX−) clusters. Any positive signal (either single or double) in the negative control was subtracted from each accepted sample. Samples with <10,000 wells qualified by the quality test were discarded and repeated. The raw dPCR data can be found in Supplementary Table 1.

### Computational and statistical analysis

For dPCR data analysis, taking “*w”* and “*n*” as the number of negative and total partitions in each reaction, respectively, the average number of target molecules per partition (λ) was calculated as λ = -ln(*w*/*n*). Taking “*v*” as the average reaction volume in each partition, total, WT and mutated DNA copies per microliter of dPCR mix were then calculated as copies/µL = λ/*v*. The mutant allele frequency (MAF) was calculated as the number of mutated copies/µL to the total number (mutated and WT) of copies/µL [25, 26].

The limit of blank (LoB) of each marker was estimated using 25 additional control samples with WT microsatellites sequences: 18 cfDNA and seven genomic DNA (gDNA). LoB was defined as the upper 95% CI of the mean false-positive MAF values [23, 27]. The limit of detection (LoD) for each marker and the reproducibility of the assays were assessed by serial dilution experiments using DNA of the positive control and cfDNA of MSI-negative patients in triplicate. LoD was defined as the lowest expected MAF exceeding the LoB with a corresponding observed positive signal in all three replicates. The experiments were carried out according to the Minimum Information for Publication of Quantitative Digital PCR Experiments (MIQE) guidelines [25], and all the required information are available in the main text or in the supplementary files.

The MAF values were considered as continuous data and bMSI was defined as the sum of the MAF values of the five markers. To facilitate the evaluation of changes in the test results in relation to surveillance round and the presence of lesion, we dichotomized the dPCR MAF data into positive (any value above the LoB) versus negative. The patients’ characteristics were summarized using basic descriptive statistics. For categorical data, frequency distribution tables were calculated, while for continuous data, descriptive statistics were estimated. Considering the baseline samples, we investigated the relationship between each marker and the presence of lesions by means of the Wilcoxon (W) or Kruskal-Wallis (KW) test depending on the nature of the variables [28]. We assessed the strength of the associations of each marker with each of the other markers using Spearman’s correlation coefficient (r_s_) and its 95% confidence interval (95% CI) calculated by the bootstrap bias-corrected and accelerated (BCa) method (95% CIBCa) [29]. Finally, we estimated the predictive capability of bMSI through a logistic model by means of the area under the receiver operating characteristic (ROC) curve (AUC). All statistical analyses were performed with the SAS software (version 9.4; SAS Institute, Inc., Cary, NC), adopting a significance level of *α* = 0.05.

## Results

### Patient characteristics and endoscopy screening outcomes

As depicted in the flow chart of Fig. 1A, the CRC surveillance study enrolled 87 LS patients with confirmed germline mutations in MMR genes and all underwent colonoscopy at baseline. Sixty-six of these patients returned at T1 and 51 at T2 for a second and third endoscopic examination. At baseline, 10 LgDs, five HgDs and three ADKs were detected by colonoscopy in 18 LS patients. At subsequent screening rounds, a lesion of each type was found in three patients at T1, while three LgDs and one HgD were detected at T2 in four patients. Except for one patient who was negative at T0 and T1 and developed an LgD at T2 (data not shown), all other lesions at T1 and T2 occurred in the 18 LS patients having a lesion at T0. None of the patients presented any other LS-related tumors during the observation period.

For the present study, all plasma samples available at the different time points from the 18 LS patients having lesions at T0 and 18 LS patients without any lesions detected during the surveillance period were considered. A total of 78 plasma samples were suitable for molecular analysis and 25 of these were collected in the presence of a colonoscopy-detected lesion: 14 LgDs, 8 HgDs, and 3 ADKs (Fig. 1B).

Overall, the median age of the patients was 56 years (interquartile range 41–62); 47% were female and 53% had germline mutations in *MLH1*, 36% in *MSH2*, and the remaining 11% in *MSH6*/*PMS2*. No differences in the distribution of sex, age, and germline mutations in MMR genes were observed between patients with and without endoscopically detected lesions at T0 (Table 1).

**Table 1:** Characteristics of Lynch syndrome (LS) patients with or without colorectal lesions at baseline (T0).

	18 LS patients with lesions at T0		18 LS patients without any lesion		*p*-value
	*N*	%	*N*	%	
Total	18	100	18	100	
Sex					
Female	7	38.9	10	55.6	0.317
Male	11	61.1	8	44.4	
Age					
Median (IQR)	58 [48–62]		56 [41–61]		0.501
MMR mutation					
MLH1	8	44.4	11	61.1	0.521
MSH2	8	44.4	5	27.8	
MSH6/PMS2	2	11.1	2	11.1	

### Set-up of drop-off dPCR assays for analysis of bMSI

**Fig. 2:**
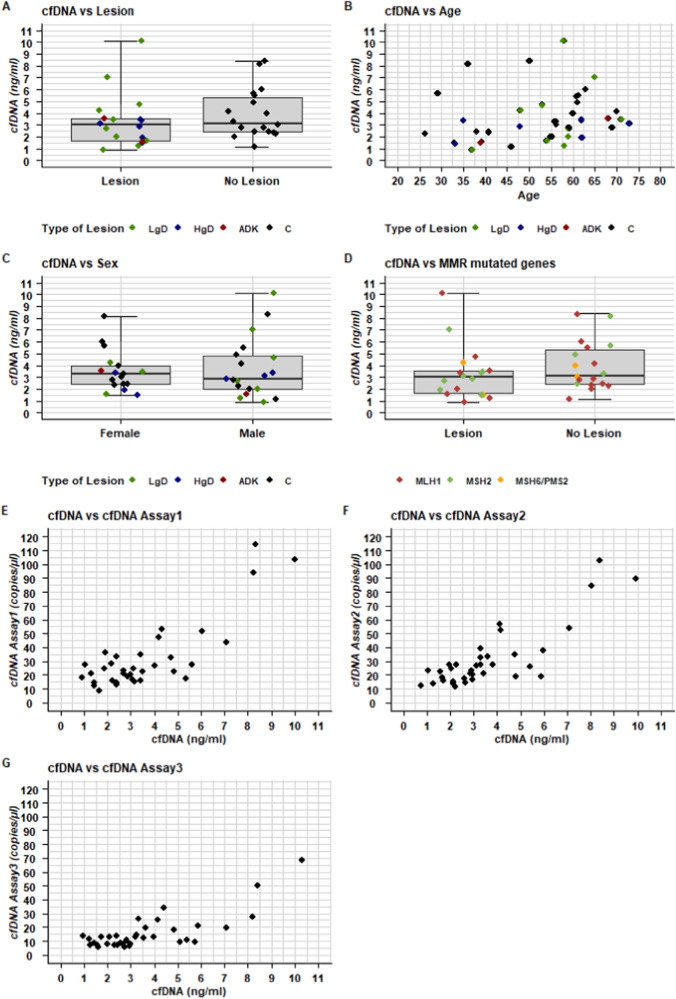
Plasma cfDNA characterization at baseline. Panels representing the distribution of cfDNA values measured by electrophoresis at the TapeStation (Agilent, Santa Clara, CA, USA) according to (**A**) lesion presence, (**B**) age, (**C**) sex, (**D**) MMR gene mutations. Each box indicates the 25th and 75th percentiles. The horizontal line inside the box indicates the median, the whiskers the extreme values measured. Individual data are represented by coloured dots according to lesion (**A**, **C**) or MMR gene mutations (**D**) details. **B** Scatter plot depicting the relationships between cfDNA (ng/mL) and age. Individual data are represented by coloured dots according to lesion details. **E**–**G** Scatter plot depicting the pairwise relationships between cfDNA (ng/mL) and each cfDNA copy assay (copies/μL) (**E**) r_s_: 0.59 (95% CIBCa: 0.32; 0.77) for Assay 1, (**F**) r_s_: 0.70 (95% CIBCa: 0.47; 0.83) for Assay 2 and (**G**) r_s_: 0.63 (95% CIBCa: 0.37; 0.79) for Assay 3.

Plasma cfDNA was extracted from 1 to 2 mL of plasma and ranged from 0.9 to 10.1 ng/mL as measured by electrophoresis. Observing the baseline plasma samples, we did not find any significant differences in cfDNA amount related to the presence of lesions (*p* = 0.289), age (rs: 0.29; 95% CIBCa: -0.05; 0.56), sex (*p* = 0.778) and MMR gene mutations (*p* = 0.684; Fig. 2A–D). The molecular analysis started from a fixed volume (6.6 µL) of elution buffer containing 0.2 ng up to 1.5 ng of cfDNA. As a result, the total amount of DNA copies/µL, whether arising from WT or mutated DNA molecules, reflected the cfDNA content in plasma samples for any of the three dPCR assays adopted (Fig. 2E) with a Spearman correlation coefficient ranging from 0.59 to 0.70.

To verify the overall performance of the assays, the LoB was first estimated using 25 WT additional samples and defined as the upper 95%CI of the mean false-positive MAF. The LoB of the five markers was 0.01% for BAT26, 0.05% for BAT25 and NR21, 0.03% for NR24, and 0.17% for Mono27 (Supplementary Fig. 1A). Afterwards, the assay reproducibility and the LoD were established by serial dilutions mixing WT and mutated DNA with expected MAFs ranging from 25% to 0.02% in triplicates. For each marker, all replicates exceeding the expected LoB had a positive signal with an rs between expected and observed MAFs higher than 0.9 (Supplementary Fig. 1B). Ultimately, the estimated LoD was 0.02% for BAT26, 0.05% for BAT25, 0.03% for NR24, 0.06% for NR21 and 0.20% for Mono27.

To further asses the reliability of the dPCR technology compared to the standard pentaplex PCR diagnostic tool, DNA extracted from two metachronous lesions occurred in a single patient diagnosed with LS was analyzed using both platforms (Supplementary Table 2). As expected, the pentaplex assay was less sensitive than dPCR in detecting MSI in tissue samples collected from precancerous and small cancerous lesions. Of note, by dPCR we were able to detected MSI in 3 and 2 loci even in the corresponding plasma sample, respectively. Representative images of the dPCR in tumor tissue and plasma samples collected from LS patients are shown in Supplementary Fig. 2.

### Association of plasma microsatellite MAF values with lesion type and patient characteristics

**Fig. 3:**
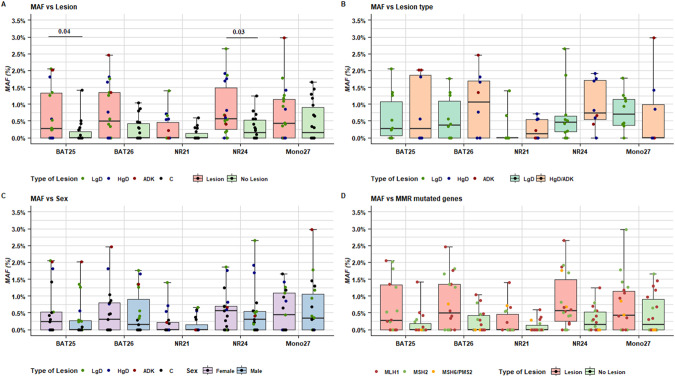
Distribution of microsatellite MAF values according to clinico-pathological characteristics. Panels representing the distribution of MAF values according to (**A**) lesion presence, (**B**) type of lesion, (**C**) sex, (**D)** MMR gene mutation at T0. Each box indicates the 25th and 75th percentiles. The horizontal line inside the box indicates the median, the whiskers the extreme values measured. Individual data are represented by colored dots according to lesion (**A**–**C**) or MMR gene mutations (**D**) details. The reported significant p-values refer to Wilcoxon test.

The MAF values of the five microsatellite markers were first analyzed in relation to each other, to the total cfDNA copies (ng/mL) as well as to patient and tumor characteristics. In detail, the MAF values of BAT26, BAT25 and NR24 were significantly higher in samples from patients with endoscopically detected lesions (Fig. 3A), but did not differ significantly between lesion types (Fig. 3B). Moreover, no statistically significant associations were found **(**Fig. 3C, D) between the distribution of each microsatellite MAF with respect to sex and MMR gene mutations.

**Fig. 4:**
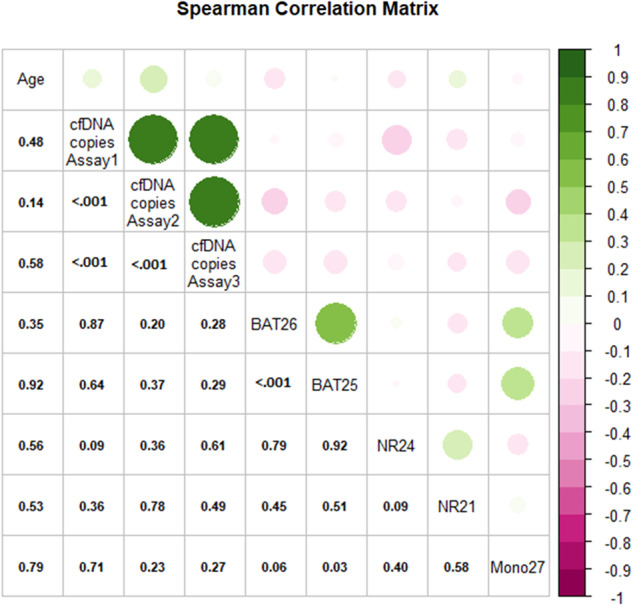
Correlogram summarizing relationships between continuous variables. Pairwise relationships in terms of Spearman correlation coefficient (*r*_s_) between continuous variables such as patients’ age, microsatellite MAF and cfDNA copies (copies/μL) of each assay are reported. Colors indicate the direction of the correlation (green for positive and pink from negative correlations, respectively) and the size of the bubble the corresponding magnitude (upper triangular matrix form); single values indicate the corresponding *r*_s_ *p*-values (lower triangular matrix form).

The correlation matrix for continuous variables represented in Fig. 4 shows that the BAT26 MAF values positively correlated with those of BAT25 (rs: 0.59; 95% CIBCa: 0.32; 0.77) and so were the Mono27 MAF values (rs: 0.37; 95% CIBCa: 0.04; 0.62). In addition, none of the MAF values correlated with age or with those of cfDNA copies detected by each assay.

**Fig. 5:**
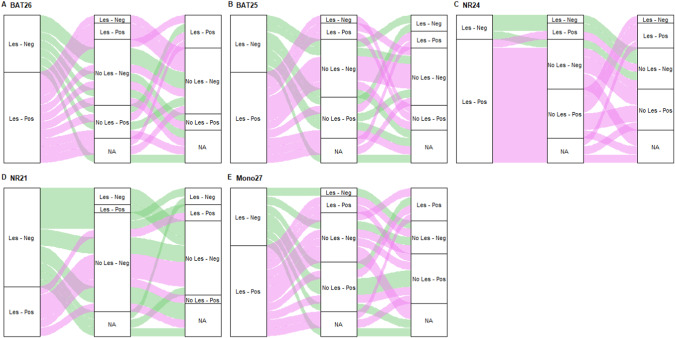
Time-trend pattern of the MAF values in the 18 LS patients with lesions at T0. **A**–**E** Sankey diagrams depicting the time trends of the five microsatellites on a dichotomized scale (i.e., negative vs positive). Colors indicates patient’s cluster profile over time according to lesion presence and markers positivity at baseline (T0).

Figure 5 and Supplementary Figure 3 illustrates the time-trend pattern of the microsatellites on a dichotomic scale (i.e., negative vs positive), considering the 18 LS patients with lesions and the 18 without lesions, respectively. Specifically, by looking at Fig. 5, if NR24 was the marker that showed the highest frequency of positive signals at each time point, NR21 was the one with the least. Except for NR21, the remaining microsatellites displayed a higher frequency of positive rather than negative signals in samples collected in presence of lesions. Notably, whether a marker is positive or not can vary over time.

### Diagnostic value of blood MSI

**Fig. 6:**
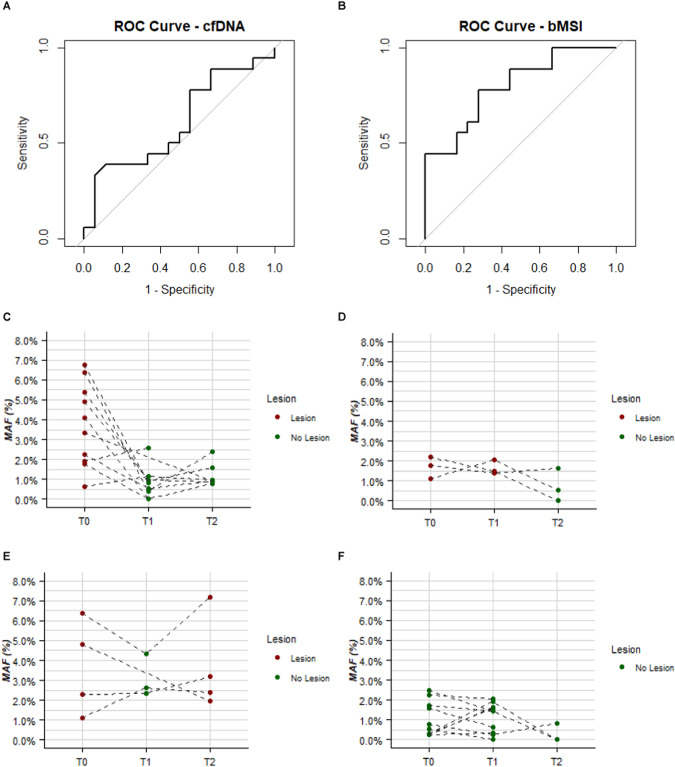
Evaluating the utility of blood MSI (bMSI) to discriminate lesion-bearing patients. ROC curves of the (**A**) cell free DNA (cfDNA) and (**B**) blood microsatellite instability (bMSI) discriminating patients with and without colorectal lesion at baseline. **C**–**F** Spaghetti plots reporting the time-trend profile of the bMSI according to patient’s cluster profile over time according to lesion presence. Dots and dashed dark gray lines indicate the patients’ values and their trend over time.

To evaluate the ability of the liquid biopsy to discriminate between samples from patients with and without lesions, we estimated the AUC for both cfDNA and bMSI on their continuous scale. While the amount of cfDNA showed no discriminatory ability with an AUC of 0.61 (95% CI: 0.42; 0.80) (Fig. 6A), bMSI was able to discriminate patients according to the presence of lesions, with an AUC of 0.80 (95% CI: 0.66; 0.94) (Fig. 6B). This result was maintained after cross-validation (AUC-CV: 0.74; 95% CI: 0.58; 0.91).

For explorative purposes, we examined bMSI in terms of sensitivity and specificity, by selecting a cutoff that guaranteed at least 75% sensitivity, the specificity of bMSI was 72%. To assess the possible utility of the tool, we estimated its positive predictive value (PPV) and negative predictive value (NPV) by considering the T0 lesion prevalence registered in our overall series: this led to a PPV of 42% and NPV of 93%.

By stratifying patients according to presence of lesions, we looked at the time-trend pattern of bMSI. In LS patients with lesions at T0, but not at T1 and T2, a decrease of bMSI values compared to the baseline was observed in 8 out of 10 (80%) patients (Fig. 6C). Conversely, in the seven patients with metachronous lesions detected at T1 (Fig. 6D) or T2 (Fig. 6E), bMSI values showed a not well defined trend over time. Lastly, when LS patients who never developed lesions were considered, the range of bMSI values at T0, T1, and T2 remained comparable (Fig. 6F).

## Discussion

This study showed for the first time that the detection of MSI in blood by dPCR was able to discriminate between LS patients with and without precancerous or cancerous colorectal lesions. The bMSI can thus be proposed as a potential biomarker in surveillance programs.

LS-related tumors are characterized by MSI, an early event already occurring in the context of precancerous lesions such as adenomas [30, 31]. Using a sensitive multiplex drop-off dPCR assay, we were able to evaluate the presence of ctDNA in plasma of patients with early lesions with allele frequencies as low as 0.01%, similar to what was previously reported for advanced CRC and breast cancer [23, 27]. Moreover, the presence of the biomarkers was not associated with the amount of starting material (in terms of cfDNA) as well as with other clinico-pathological characteristics such as age, sex and germline mutational status of MMR genes. The advantages provided by combining multiplex assays further allow us to save starting plasma samples, while the use of drop-off probes makes it possible to calculate the frequency of mutated alleles by comparing single and double positive signals [32].

The debate about the issue of endoscopic surveillance frequency is very active in the scientific community [5, 18]. It is well established that the risk of cancer differs among LS patients based on the germline-altered MMR gene (*MLH1*, *MSH2*, *MSH6,* or *PMS2*), implying that quality of colonoscopy, surveillance intervals, treatment, and preventive strategies may differ between LS subgroups [33]. However, a significant proportion of LS patients still develop interval CRC despite regular colonoscopies, even when performed annually [7, 18]. Differences in the classic adenoma-carcinoma sequence for CRC development, leading to fast progression of adenomas and/or adenoma-free progression to cancer, likely from MMR-deficient crypt foci, are the main causes of this lack of efficacy [6, 7].

Another concern, is the high frequency of metachronous tumors developing in these individuals [15]. In the present cohort, 37% of patients developed metachronous lesions during the study duration. Although the results are very preliminary, the time-course analysis shows that, in absence of lesions, patients who develop metachronous lesions tend to have a higher bMSI than patients for whom no lesions were observed. This observation, if confirmed, suggests that bMSI monitoring might be useful for surveillance purposes.

The very promising but still suboptimal accuracy of the predictive model here reported allows us to give a first indication of its actual use. The high NPV leads us to favor performance characteristics, such as sensitivity to obtain better results in terms of clinical applicability. In future studies, we will therefore seek to further maximize sensitivity and NPV to safely extend the intervals between endoscopic rounds in low-risk participants.

We acknowledge that this study has some limitations, mainly due to its retrospective design. One of the main technical issues was the small amount of plasma available (≤2 mL) as compared with other studies, which recommended using at least 3 mL of plasma [34]. We preferred to use these limited samples to assess the utility of combining all markers, leaving the assessment of the technical reproducibility, including partitioning statistics, of the assays to the set-up phase, where we used control samples with larger starting volumes. Furthermore, it was possible to determine the presence or absence of the bMSI signal in relation to the endoscopic lesion but not the timing of the onset of the signal. Finally, although MSI is a peculiarity of all LS-related malignancies, the fact that none of the analyzed LS patients developed other tumors besides CRC did not allow us to evaluate the efficacy of bMSI to the surveillance of other organs.

To overcome these limitations, a prospective observational surveillance study in collaboration with the Italian Association of Patients with Hereditary Digestive Tract Cancers (APTEAD) was recently initiated at the National Cancer Institute (Fondazione IRCCS Istituto Nazionale dei Tumori) in Milan. Enrolled LS patients will undergo annual endoscopic examination and all routine check-ups for LS surveillance. Plasma samples will be collected at shorter intervals (between surveillance colonoscopies) to assess bMSI. This prospective trial aims to refine the methodological workflow, consolidate the preliminary results of the present retrospective study, and clarify the interval between the marker signal and the onset of adenoma or CRC.

### Supplementary information

Supplementary Legends

Supplementary Table 1

Supplementary Table 2

Supplementary Figures

## Data Availability

The raw data are available as supplementary files.
