# Supplementary material for: Detection of (pre)cancerous colorectal lesions in Lynch syndrome patients by microsatellite instability liquid biopsy
Source: Cancer Gene Ther. 2024 Feb 9;31(6):842–50. doi: 10.1038/s41417-023-00721-z (PMC11192631; doi:10.1038/s41417-023-00721-z)
Supplement: Supplementary file 1 — Supplementary Legends [file 41417_2023_721_MOESM1_ESM.docx]

**Supplementary Figures’ Legends**

**Supplementary Figure 1. Evaluating the performance of the digital PCR assays.** Assessment of (**A**) the limit of blank (LoB) and (**B**) the limit of detection (LoD) of the five markers. LoB was defined as the upper 95% confidence interval of the mean false-positive MAF values of 25 wild-type DNA samples. LoD was determined by serial dilution experiments using MSI-positive and negative DNA in triplicate and defined as the lowest expected MAF exceeding the LoB with a corresponding observed positive signal in all three replicates. Means with standard deviations are reported.

**Supplementary Figure 2**. **Representative images of dPCR output for the three dPCR assays**. Images were generated using the QuantStudio 3D AnalysisSuite tool (ThermoFisher). The respective MAF values are reported under each image.

**Supplementary Figure 3**.  **Time-trend pattern of the MAF values in the 18 LS patients without lesions at any time points. (A-E)** Sankey diagrams depicting the time trends of the five microsatellites on a dichotomized scale (i.e., negative vs positive). Colors indicates patient’s cluster profile over time according to lesion presence and markers positivity at baseline (T0).

**Supplementary Tables’ Legends**

**Supplementary Table 1:** Clinico-pathological characteristics of patients with Lynch syndrome and raw digital PCR data.

**Supplementary Table 2**: Microsatellites’ instability in tissue and plasma samples evaluated by pentaplex polymerase chain reaction (PCR) and digital PCR (dPCR).
