# Supplementary material for: Detection of (pre)cancerous colorectal lesions in Lynch syndrome patients by microsatellite instability liquid biopsy
Source: Cancer Gene Ther. 2024 Feb 9;31(6):842–50. doi: 10.1038/s41417-023-00721-z (PMC11192631; doi:10.1038/s41417-023-00721-z)
Supplement: Supplementary file 3 — Supplementary Table 2 [file 41417_2023_721_MOESM3_ESM.docx]

**Supplementary Table 2:** Microsatellites’ instability in tissue and plasma samples evaluated by pentaplex polymerase chain reaction (PCR) and digital PCR (dPCR).

| Lesion | Sample | Platform | BAT26 | BAT25 | NR24 | NR21 | Mono27 | NR22 |
| --- | --- | --- | --- | --- | --- | --- | --- | --- |
| HgD | Tissue | Pentaplex PCR | Instability | Stability | Stability | Stability | NA | Stability |
|  | Tissue | dPCR | Instability | Instability | Instability | Instability | Instability | NA |
|  | Plasma | dPCR | Instability | Instability | Stability | Stability | Stability | NA |
|  |  |  |  |  |  |  |  |  |
| ADK | Tissue | Pentaplex PCR | Instability | Stability | Stability | Stability | NA | Stability |
|  | Tissue | dPCR | Instability | Instability | Instability | Stability | Instability | NA |
|  | Plasma | dPCR | Instability | Instability | Instability | Stability | Stability | NA |
