# Supplementary figures and images for: Detection of (pre)cancerous colorectal lesions in Lynch syndrome patients by microsatellite instability liquid biopsy
Source: Cancer Gene Ther. 2024 Feb 9;31(6):842–50. doi: 10.1038/s41417-023-00721-z (PMC11192631; doi:10.1038/s41417-023-00721-z)

## Slide 1
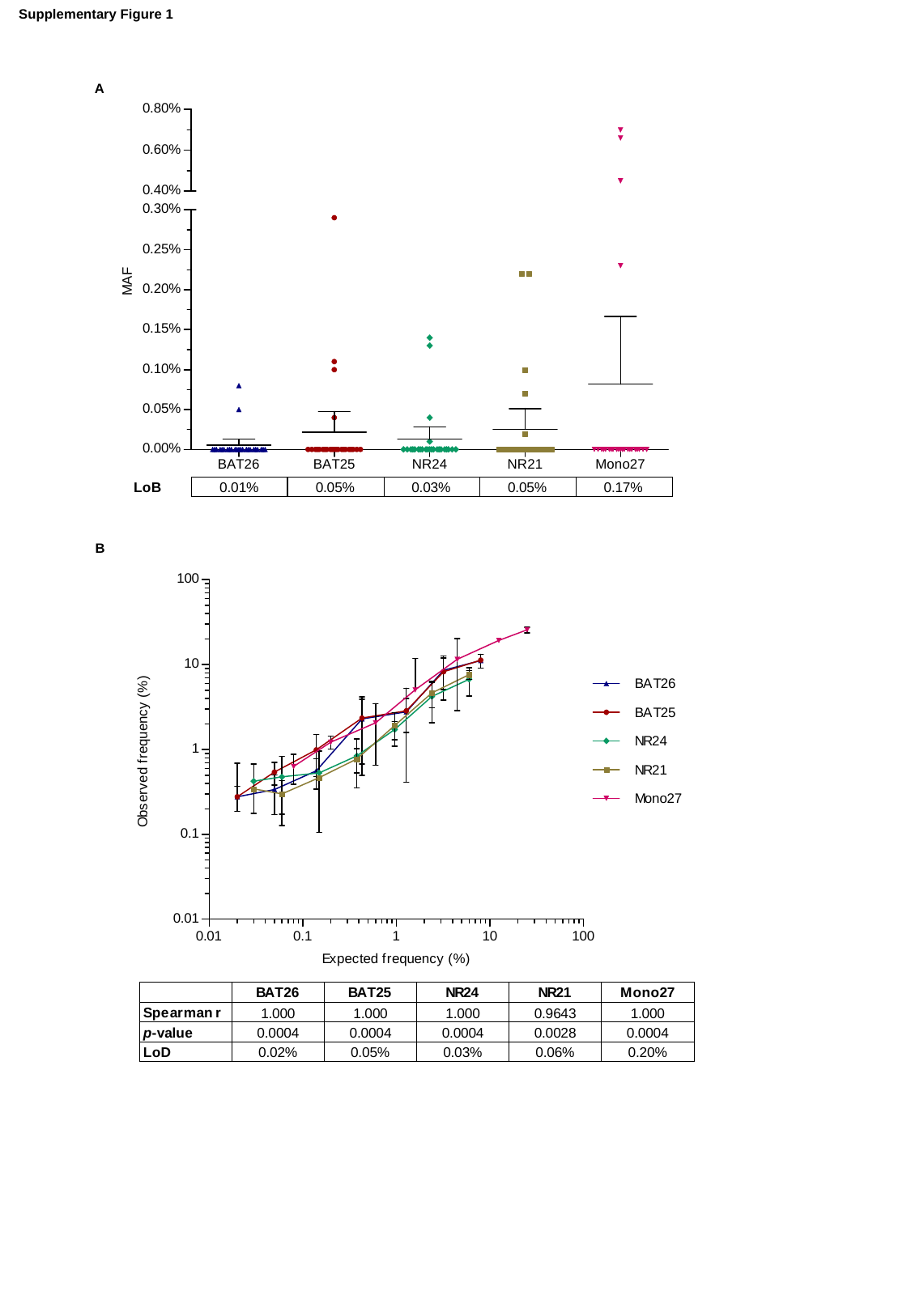

Supplementary Figure 1
A
B

## Slide 2
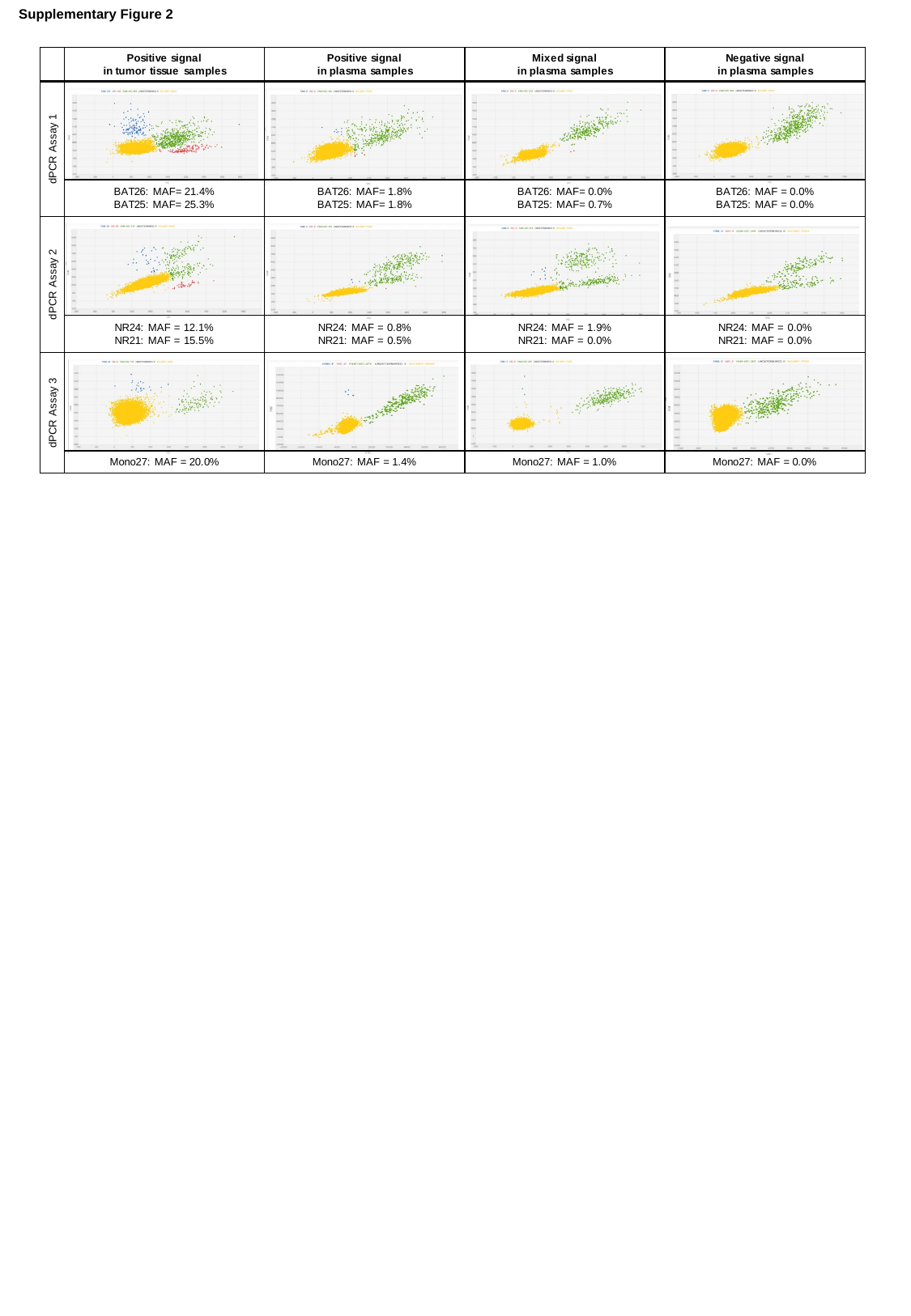

Supplementary Figure 2

## Slide 3
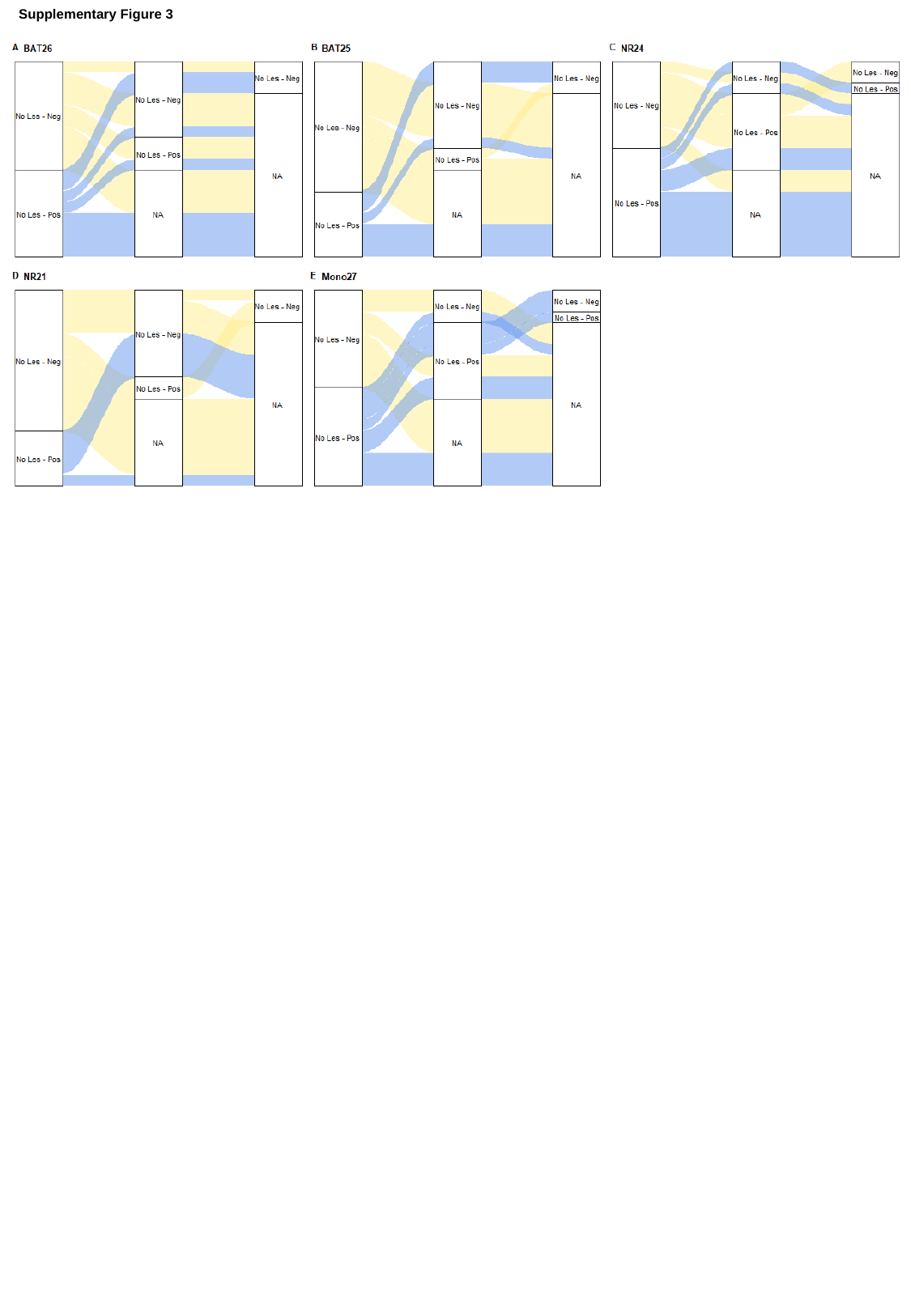

Supplementary Figure 3

Supplement: Supplementary file 4 — Supplementary Figures [file 41417_2023_721_MOESM4_ESM.pptx]
